# Gut Microbiotas, Plasma Metabolites, and Autism Spectrum Disorder: A Bidirectional Mendelian Randomization Analysis

**DOI:** 10.3390/pathogens14111137

**Published:** 2025-11-10

**Authors:** Jiayi Zhou, Zhang Fu, Yunfei Gao, Caiyan An, Zhiqiang Zhang, Xin Zhong, Liusuyan Tian, Xiuyan Yang, Junjing Zhang, Qingyuan Zhang, Dilong Wang, Ningning Li

**Affiliations:** 1 Tomas Lindahl Nobel Laureate Laboratory, The Seventh Affiliated Hospital of Sun Yat-sen University, Shenzhen 518107, China; zhoujiayi2018@126.com (J.Z.); fuzhang1@sysush.com (Z.F.); zhangzhq79@mail2.sysu.edu.cn (Z.Z.); zhongx69@mail2.sysu.edu.cn (X.Z.); tianlsy@mail2.sysu.edu.cn (L.T.); yanzihaida@163.com (X.Y.); 2 Department of Geriatrics, The Seventh Affiliated Hospital of Sun Yat-sen University, Shenzhen 518107, China; 3 Department of Otolaryngology, The Seventh Affiliated Hospital of Sun Yat-sen University, Shenzhen 518107, China; gaoyf25@mail.sysu.edu.cn; 4 Inner Mongolia Key Laboratory of Allergic Diseases, Foundational and Translational Medical Research Center, Hohhot First Hospital, Hohhot 010030, China; acy_1999@163.com; 5 Digestive Diseases Center, Guangdong Provincial Key Laboratory of Digestive Cancer Research, The Seventh Affiliated Hospital of Sun Yat-sen University, Shenzhen 518107, China; 6 Department of Pediatrics, Sun Yat-sen Memorial Hospital, Sun Yat-sen University, Guangzhou 510120, China; 7 Future Medical Center, Shenzhen University of Advanced Technology, Shenzhen 518107, China

**Keywords:** ASD, gut microbiota, plasma metabolites, mendelian randomization

## Abstract

**Background**: Previous studies have indicated that the gut microbiome and plasma metabolites play key roles in autism spectrum disorder (ASD), but their causal relationships remain unclear. Linkage disequilibrium score regression (LDSC) and Mendelian randomization (MR) are powerful tools for assessing genetic causality. This study uses LDSC and MR to investigate the genetic links between the gut microbiome and ASD and explore the mediating role of plasma metabolites. **Methods**: To explore the genetic relationships between the gut microbiome, plasma metabolites, and ASD, we obtained summary statistics from large-scale genome-wide association studies (GWAS). Gut microbiome data came from a MiBioGen consortium meta-analysis (*N* = 18,340), ASD data from the Danish Psychiatric Central Research Register (DPCRR) (*N* = 18,382), and plasma metabolite data from the Canadian Longitudinal Study of Aging (CLSA) *(N* = 8299). We applied LDSC and bidirectional MR to analyze the genetic associations between the gut microbiome and ASD and plasma metabolites and ASD. Mediation MR was used to assess the mediating role of plasma metabolites in the gut microbiome-ASD relationship. **Results**: LDSC analysis revealed significant genetic correlations between the gut microbiota *Lachnospiraceae* NK4A136 group and *Sellimonas* with ASD. Moreover, bidirectional MR demonstrated causal effects of five gut microbial genera on ASD risk, as indicated by inverse variance weighted (IVW) methods. Similarly, we identified 49 plasma metabolites that exhibited genetic correlations with ASD, and 58 metabolites had causal effects on ASD in MR analysis. Mediation analysis revealed that specific bacteria, *Ruminiclostridium5*, reduce the occurrence of ASD through metabolites Delta-CEHC and Docosadioate (C22-DC). Furthermore, *Ruminococcaceae UCG005* and *Sutterella* modulate ASD by inhibiting Serotonin and N-acetyl-L-glutamine, respectively. **Conclusions**: This study provides evidence of a causal relationship between the gut microbiome and ASD, with plasma metabolites acting as a potential mediator. Our findings offer new insights into the causal mechanisms linking the gut microbiome and ASD and provide a theoretical foundation for microbiome-based therapeutic strategies.

## 1. Introduction

Autism Spectrum Disorders (ASD) encompass a range of complex neurodevelopmental disorders characterized by significant impairments in social interaction, communication, and the presence of restrictive and repetitive behaviors [1]. The prevalence of ASD has been steadily rising, with recent estimates indicating that approximately 1 in 43 children worldwide (2.30%) are affected [2], underscoring the urgency of addressing this public health issue. The clinical manifestations of ASD typically become apparent by the age of three years; milder cases are often overlooked, resulting in delayed diagnosis and intervention [3]. Early identification and intervention are crucial for enhancing outcomes in areas such as social functioning, language development, and adaptive behavior.

Despite substantial progress in understanding the epidemiology of ASD, its precise etiology remains incompletely understood. Current research reveals that ASD arises from a complex interplay of genetic and environmental factors. Genetic studies have identified several mutations, including those in the SHANK3 [4] and CNTNAP2 [5] genes, which are implicated in ASD pathophysiology. In addition, prenatal exposure to environmental risk factors such as pollutants, teratogens, and maternal nutritional deficiencies has been linked to an increased risk of ASD [6,7,8]. However, although both genetic and environmental factors play critical roles in the development of ASD, they do not fully account for the observed heterogeneity in ASD prevalence, with significant variation remaining among individuals with similar genetic and environmental backgrounds.

Microbes residing in symbiosis within the human gastrointestinal tract play a pivotal role in health and disease [9]. Increasing evidence shows that the gut microbiota of individuals with ASD differs significantly from that of healthy controls, with marked changes in the abundance of specific bacterial taxa [10]. Pathogenic species such as Bacteroides and Fusobacterium are often elevated in ASD [11], while beneficial bacteria like *Bifidobacterium* and *Lactobacillus* are reduced [12]. Our own work in the 16p11.2^dp/+^ autism mouse model has similarly shown decreased levels of *Faecalibaculum* and *Romboutsia* and increased levels of *Turicibacter* and *Prevotellaceae UCG_001* [13]. These microbiome alterations may influence ASD pathogenesis via the gut–brain axis, potentially through modulation of short-chain fatty acids, immune dysregulation, or disruption of intestinal barrier integrity (e.g., “leaky gut”) [14,15,16]. Despite a growing body of evidence linking the microbiota to ASD, the causal relationship remains unclear. Establishing causality could pave the way for microbiome-targeted interventions and provide critical insights into neurodevelopmental disorders.

Plasma metabolites, small molecules produced through metabolic pathways, reflect physiological states and may serve as biomarkers for disease. In ASD, plasma metabolite profiles differ significantly from those in healthy controls, particularly in amino acids, fatty acids, neurotransmitters, and other metabolites [17]. Notable examples include altered levels of tryptophan and its derivatives (e.g., serotonin) [18], phenylalanine [19], glutamate [20], γ-aminobutyric acid (GABA) [21], bile acids (e.g., deoxycholic acid) [22], and fatty acids such as omega-3 (n-3) and omega-6 [23]. These changes may be key players in ASD pathogenesis. Plasma metabolites are hypothesized to mediate ASD through mechanisms involving gut microbiota and environmental influences. For example, gut-derived short-chain fatty acids (SCFAs), including propionate and butyrate, modulate immune responses and neurodevelopment, correlating with ASD symptoms [11]. Similarly, shifts in tryptophan metabolism may impact brain development and behavior, driving ASD progression [18]. These metabolites not only offer potential as biomarkers but also provide insights into the complex mechanisms underlying ASD. However, most evidence is correlational, and causal roles for plasma metabolites in ASD remain to be established. Determining these causal relationships could uncover novel therapeutic targets and deepen understanding of ASD’s underlying mechanisms.

Current observational studies are insufficient to definitively determine the causal relationships between gut microbiota, plasma metabolites, and ASD, making further exploration essential. Statistical methods based on genome-wide association studies (GWAS) offer promising tools for such investigations. Linkage disequilibrium score regression (LDSC) uses GWAS summary statistics to evaluate genetic correlations while avoiding sample overlap bias [24]. Mendelian randomization (MR), leveraging genetic variants as instrumental variables, has emerged as a powerful approach for inferring causality [25]. Since genotypes precede phenotypes and alleles are randomly assigned at conception, MR minimizes biases from measurement error, confounding, and reverse causation.

In this study, we applied LDSC and MR to evaluate the genetic correlations and causal relationships between genetically predicted gut microbiota, plasma metabolites, and ASD. We further investigated whether plasma metabolites mediate the pathway from gut microbiota to ASD. Additionally, through reverse causality analysis, we explored whether genetic predisposition to ASD influences gut microbiota and plasma metabolites. The key findings of our study, which are highlighted as follows, directly address these objectives:

1. Identified causal links between gut microbiota, plasma metabolites, and ASD using Mendelian Randomization.
2. Revealed key taxa, including *Ruminiclostridium5*, *RuminococcaceaeUCG-005*, and *Sutterella*, influencing ASD through metabolites.
3. Highlighted metabolites like serotonin, N-acetyl-L-glutamine, Delta-CEHC, and Docosadioate (C22-DC) as mediators in the gut–brain axis.
4. Proposed diagnostic and therapeutic targets for ASD based on gut microbiota and metabolites.

## 2. Method

### 2.1. Study Design Overview

**Figure 1:**
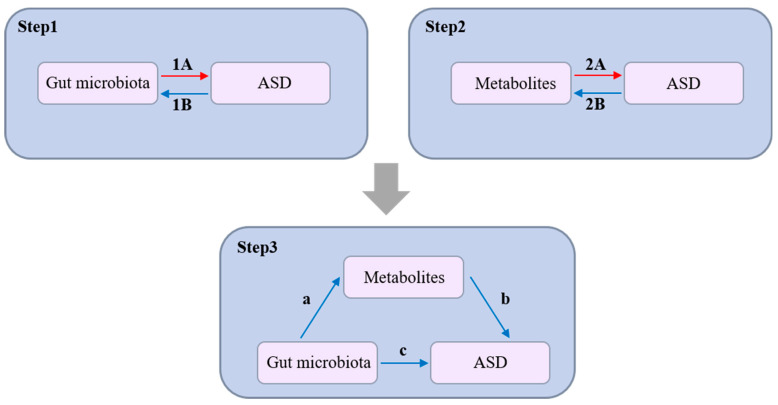
Study overview. **Step 1**: Assessment of the causal effects of 211 gut microbiota taxa on ASD using Single Nucleotide Polymorphisms (SNPs) as instrumental variables (IVs). 1A: Represents a positive causal relationship of gut microbiota on ASD. 1B: Represents a negative causal relationship of gut microbiota on ASD. **Step 2**: Investigation of the impact of 1458 plasma metabolites on ASD, utilizing SNPs as IVs to identify key metabolic pathways potentially contributing to ASD pathogenesis. 2A: Represents a positive causal relationship of metabolites on ASD. 2B: Represents a negative causal relationship of metabolites on ASD. **Step 3**: Mediation analysis to evaluate the role of plasma metabolites as intermediaries in the association between gut microbiota and ASD, providing insights into the involvement of the gut–brain axis in ASD development. a (Path a): Represents the causal effect of gut microbiota on the mediating metabolites. b (Path b): Represents the causal effect of the mediating metabolites on ASD after controlling for the influence of gut microbiota. c (Path c): Represents the direct effect of gut microbiota on ASD (not through that specific metabolite).

In our study, we utilized a Mendelian Randomization (MR) approach to assess the potential causal links between gut microbiota, plasma metabolites, and Autism Spectrum Disorder (ASD), outlined in a comprehensive three-step process (Figure 1). Initially, we analyzed the impact of 211 types of gut microbiota on ASD (Step 1), employing Single Nucleotide Polymorphisms (SNPs) as instrumental variables (IVs) to establish a causal connection. Subsequently, our examination extended to the potential influences of 1458 plasma metabolites on ASD (Step 2), using SNPs as IVs to pinpoint crucial metabolic pathways potentially contributing to ASD’s etiology. The final phase involved a mediation analysis (Step 3), which assessed the role of plasma metabolites as intermediaries in the link between gut microbiota and ASD, thereby elucidating the gut–brain axis’s involvement in ASD. This methodological framework relies on three foundational assumptions: that the SNPs serving as IVs are significantly related to the exposures (either gut microbiota or metabolites); these IVs are free from associations with any confounders; and the IVs affect the ASD outcome exclusively through these exposures, without a direct impact. The entire study protocol is detailed in the Supplementary Figure S1.

### 2.2. Data Sources and Instruments

#### 2.2.1. Gut Microbiota

Genetic instruments for gut microbiota were sourced from the MiBioGen consortium (https://mibiogen.gcc.rug.nl (accessed on 15 January 2024)), which gathered data on the fecal 16S rRNA sequencing data and GWAS information from 18,340 participants across 24 cohorts, 78% of whom were of European descent. Among these cohorts, twenty-two cohorts comprised adult or adolescent individuals (*N* = 16,632), and two cohorts consisted of children (*N* = 1708). After excluding unclassifiable microbial taxa, a total of 211 taxa were identified, including 131 genera, 35 families, 20 orders, 16 classes, and 9 phyla. GWAS data from all cohorts were adjusted for covariates such as genetic principal components, age, sex, and other relevant factors. We identified SNPs meeting the genome-wide significance threshold (*p* < 5 × 10^−8^) in these studies to serve as genetic instruments. Subsequent check in the GWAS Catalog confirmed that the underlying studies for these SNP-microbiota associations had pre-adjusted their models for covariates (e.g., age, sex), thereby obviating the need for additional correction on our part.

#### 2.2.2. Plasma Metabolome

In this study, serum metabolite data were derived from the Canadian Longitudinal Study of Aging (CLSA) study (PubMed: 31633757). After excluding 203 European individuals with first- and second-degree relatives identified through kinship inference, we obtained a cohort of 8299 participants of European ancestry (doi: 10.1038/s41588-022-01270-1). Post-rigorous GWAS quality control, approximately 15.4 million SNPs were included in the analysis. Plasma metabolites were quantified using UPLC-MS/MS, identifying 1458 metabolites. Following a series of standardization and quality control steps, which eliminated non-compliant data, a final selection of 1091 metabolites (including 850 known substances and 241 unknown entities) and 309 metabolite ratios were chosen for genome-wide association analysis. Given that metabolites serve both as substrates and products of enzymatic reactions, investigating the genetic underpinnings of their ratios enhances our understanding of broader biological processes; hence, the analysis also encompassed 309 metabolite ratios. We identified single nucleotide polymorphisms (SNPs) meeting the genome-wide significance threshold (*p* < 1 × 10^−5^) in these GWASs to serve as genetic instruments.

#### 2.2.3. ASD

The Genetic data for ASD patients were sourced from the Danish Psychiatric Central Research Register (DPCRR) (PubMed: 21775352), incorporating 18,382 child cases diagnosed with ASD by psychiatrists according to ICD10 in 2013 or earlier, along with 27,969 control children excluding an ASD diagnosis. Through rigorous GWAS criteria screening, a total of 9,112,386 SNPs were filtered for genetic studies.

#### 2.2.4. Statistical Analysis

To ensure the independence and validity of our IVs, we first conducted an association analysis, selecting SNPs strongly associated with the exposure factor from GWAS based on a *p*-value criterion of less than 1 × 10^−5^. Additionally, we removed SNPs in linkage disequilibrium (LD) by setting a threshold of R^2^ less than 0.001 within a 10,000 KB range, enhancing the independence of our IVs. To ensure the strength of our instrumental variables, we further excluded weak instruments with an F-statistic value of less than 10.

#### 2.2.5. Genetic Correlation Analysis

We used LDSC to estimate the genetic correlation (rg) between gut microbiota/blood metabolites and ASD. Initially, GWAS summary statistics were filtered based on the HapMap3 reference data, excluding non-SNPs and SNPs with MAF < 0.01. LDSC quantifies the true polygenic signal or bias by examining the association between test statistics and linkage disequilibrium, allowing for genetic correlation assessment from GWAS summary statistics, independent of sample overlap. The product of z-scores for each variant from Trait 1 and Trait 2 was regressed against the LD score to estimate genetic covariance, normalized by SNP heritability. A significance threshold of *p* < 0.0004 (Bonferroni correction: 0.05/119) was considered statistically significant, while 0.0004 < *p* < 0.05 indicated potential evidence for potential genetic correlation.

#### 2.2.6. Two-Sample MR

Our primary MR analysis method was IVW, supplemented with MR-Egger, Weighted median, Weighted mode, Simple mode methods, and heterogeneity was assessed using IVW and MR-Egger methods (*p* < 0.05). The MR-Egger method was specifically employed to detect unknown horizontal pleiotropy (*p* < 0.05), where a non-zero intercept indicates potential bias. To further mitigate potential pleiotropic effects, a leave-one-SNP-out approach was utilized for assessment. All analyses were conducted in the R environment using the TwoSampleMR package (version 0.5.7), which facilitated the harmonization of exposure and outcome datasets, including phenotypes, effect alleles, effect allele frequencies, effect sizes, and standard errors for each SNP. Effect estimates are reported as beta values for continuous outcomes and ORs (95% confidence intervals) for binary outcomes. Bonferroni correction was applied to adjust the *p*-value threshold for multiple comparisons, reducing the risk of false positives.

#### 2.2.7. Median MR

To determine whether blood metabolites mediate the effect of gut microbiota on ASD, we divided the total effect of gut microbiota on ASD into direct (non-mediated) and indirect (mediated) components. We employed a two-step MR strategy, also known as the product of coefficients method, to assess mediators individually. The first step involved estimating the impact of gut microbiota on mediators (blood metabolites) using univariable MR. The second step assessed the effect of mediators on ASD. The indirect effect was calculated by multiplying the estimates from both analytical steps. The direct effect is the total effect of gut microbiota on ASD minus the indirect effect. The mediation proportion was determined as the ratio of the indirect effect to the total effect, with 95% confidence intervals.

## 3. Result

### 3.1. Cause Effects of Gut Microbiota on ASD

#### 3.1.1. LDSC Analysis

**Figure 2:**
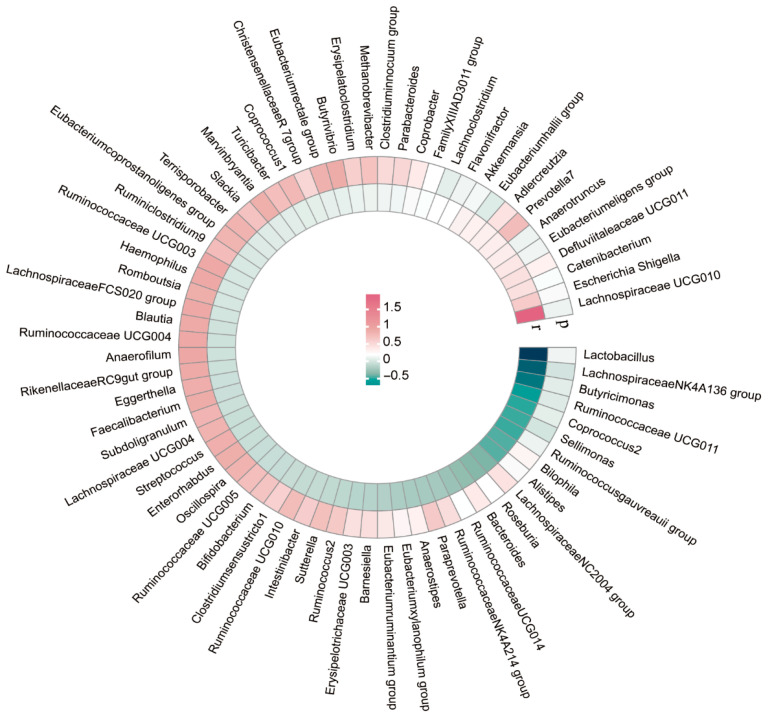
Circular heat map of genetic correlations between gut microbes and ASD.

We conducted LDSC analysis to assess the genetic correlations between 131 genus-level gut microbiota and ASD. Due to constraints such as low heritability and limited sample sizes, not all genera were suitable for the analysis. Ultimately, genetic correlation estimates were obtained for 74 genera in relation to ASD. As depicted in Table 1 and Figure 2, the LDSC analysis indicated a significant correlation between the *Lachnospiraceae NK4A136 group* and ASD (rg = −1.196, *p* = 0.005), as well as between *Sellimonas* and ASD (rg = −0.467, *p* = 0.005). Comprehensive details on all genetic correlation findings are provided in Supplementary Table S1.

#### 3.1.2. Two-Sample MR and Sensitivity Analysis

Following our screening criteria, 1531 SNPs were identified as IVs for 119 genus-level gut microbiotas at a significance threshold of *p* < 1 × 10^−5^. Then, all 1531 instrumental SNPs were subjected to a phenome-wide association study (PheWAS) screen via the GWAS Catalog to assess potential horizontal pleiotropy. Using an automated script, we batch-queried all SNPs and excluded any that were genome-wide significantly associated (*p* < 5 × 10^−8^) with pre-specified potential confounding traits, including age. Following this rigorous screening, all 1531 SNPs were retained for the final Mendelian randomization analysis. The F-statistics for selected IVs all exceeded 10, indicating a minimal risk of weak instrument bias. Seven genus-level gut microbiotas demonstrated potential associations with ASD in IVW MR analyses, each supported by more than four SNPs. Subsequent analyses, including the radial MR-Egger intercept and MR-PRESSO global tests, revealed evidence of pleiotropy for *Faecalibacterium* (*p* = 0.04). Ultimately, six genus-level gut microbiotas—*Turicibacter*, *Ruminococcaceae* UCG005, *Sutterella*, *Ruminiclostridium5*, *Ruminococcus1*, and *Dorea*—were identified as having genetic correlations with ASD, as shown in Table 2. Detailed scatter plots and sensitivity analyses are provided in Supplementary Figures S2 and S3, respectively. In summary, we identified six gut bacterial genera that exhibit significant genetic correlations with ASD, implying that these gut microbiotas may play a causal role in the development of ASD. From a genetic perspective, variations in these bacterial communities could increase the risk of ASD.

#### 3.1.3. Reverse MR Analysis

In accordance with our established screening protocol, we identified 1476 SNPs as IVs for an analysis involving 18,382 individuals diagnosed with ASD, each SNP surpassing the pre-specified significance level of *p* < 1 × 10^−5^. The F-statistics for selected IVs all exceeded 10, indicative of a negligible likelihood of bias due to weak instruments. We employed five distinct MR methods to assess the influence of ASD on the relative abundance of six targeted bacterial genera. The analysis, particularly through the IVW method, yielded no statistically significant outcomes (Table 3). The results of the reverse MR analysis did not demonstrate a significant causal effect of ASD on these six gut bacterial genera, indicating that ASD cannot be considered the cause of changes in these specific microbial communities. Therefore, our findings support a unidirectional causal relationship, where these six gut bacterial genera may increase the risk of ASD by influencing an individual’s genetic background, while ASD itself is not the cause of these changes in the gut microbial communities. These results highlight the potential key role of gut microbiota in the pathogenesis of ASD and imply that modulation of the gut microbiome could be a potential strategy for the prevention or treatment of ASD in the future.

### 3.2. Cause Effects of Plasma Metabolites on ASD

#### 3.2.1. LDSC Analysis

We performed LDSC analysis to evaluate the genetic correlations between 1400 plasma metabolites and ASD. Given constraints such as genetic heterogeneity, the complexity of metabolic pathways, and the influence of environmental factors, not all metabolites were suitable for this analysis. Ultimately, we derived genetic correlation estimates for 1170 metabolites in relation to ASD. After screening (*p* < 0.05), a total of 49 metabolites exhibited correlations with ASD. Among these, negative correlations included Salicylate to Caprylate (8:0) ratio and Cytidine to N-acetylglucosamine to N-acetylgalactosamine ratio among 34 metabolites/metabolite ratios. Positive correlations included Hexanoylglycine and Cysteinylglycine to Taurine ratio among 15 metabolites/metabolite ratios (Table 4).

#### 3.2.2. Two-Sample MR and Sensitivity Analysis

**Figure 3:**
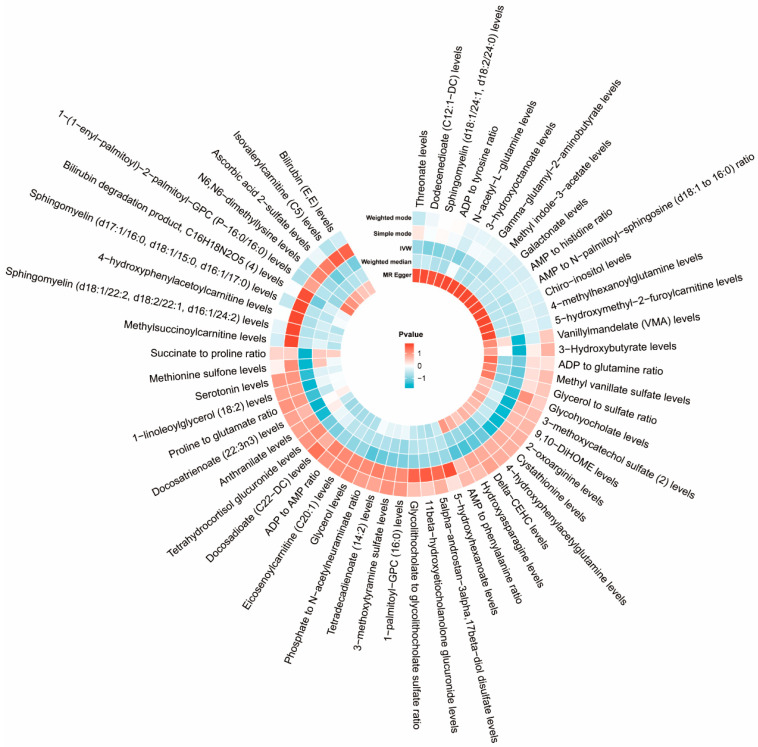
Circular heat map of MR results for causal associations between plasma metabolites and ASD.

Following our established screening criteria, 34,843 SNPs were identified as IVs for 1458 plasma metabolites at a significance threshold of *p* < 1 × 10^−5^. The F-statistics for selected IVs all above 10, indicating a low likelihood of weak instrument bias. Fifty-eight metabolites showed potential associations with ASD in IVW MR analyses, each supported by more than four SNPs. Further analyses, including the radial MR-Egger intercept and MR-PRESSO global tests, revealed no pleiotropy among these 58 metabolites. In conclusion, we identified 58 plasma metabolites with significant genetic correlations with ASD, indicating that these metabolites might play a causal role in ASD development (as depicted in Figure 3 and detailed in Supplementary Table S1). Genetically, variations in these metabolite profiles could elevate the risk of ASD.

#### 3.2.3. Reverse MR Analysis

Utilizing the same extraction methodology consistent with our ASD analysis, we applied five distinct MR methods to evaluate the impact of ASD on the relative abundance of 58 plasma metabolites. The analysis, especially via the IVW method, revealed significant changes in the metabolites 11beta-hydroxyetiocholanolone glucuronide and 9,10-DiHOME (Table 5 and Supplementary Table S2). The outcomes of the reverse MR analysis demonstrate that ASD cannot be deemed the cause of alterations in these specific metabolites. Hence, assuming a reciprocal causality between 11beta-hydroxyetiocholanolone glucuronide and 9,10-DiHOME metabolites and ASD is untenable and contradicts Mendelian principles. Accordingly, we conclude that aside from these two metabolites, the remaining 56 metabolites have a genetic causal relationship with the incidence of ASD. These findings underscore the pivotal role that plasma metabolites may play in the etiology of ASD and propose that modulating these metabolites could serve as a potential approach for the future prevention or treatment of ASD.

### 3.3. Causal Effects of Cardiometabolic Diseases on Gut-Dependent Metabolites

#### Median MR

**Figure 4:**
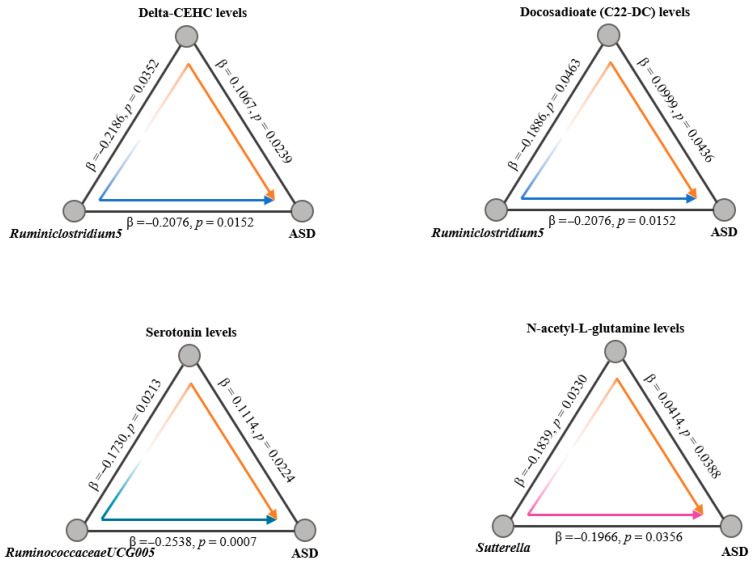
Two-step Mendelian randomization analyses of the causal effects between gut microbes, plasma metabolites and ASD. Arrow colors denote the direction and significance of causal effects: blue arrows represent significant negative causal effects; orange arrows represent significant positive causal effects; the purple arrow indicates a non-significant association. The β estimates reflect the change in the outcome (in SD units) per unit increase in the exposure. Color saturation of significant paths is proportional to the absolute magnitude of the β estimate.

Given the hypothesized influence of serum metabolites on the progression from gut microbiota alterations to ASD, we employed a two-step mediation MR framework for our analysis. Initially, we conducted a two-sample MR study to explore the associations between the gut microbiome and plasma metabolites. By utilizing the same extraction methodology as applied in our analysis of the gut microbiota, we leveraged five distinct MR methods to assess the impact of gut microbiota composition on the relative abundance of 56 plasma metabolites. Our sensitivity and pleiotropy assessments for these metabolite associations revealed no significant discrepancies. Cross-referencing the positive findings from our previous MR analyses linking specific bacteria with ASD, we identified intersections involving 5 bacterial taxa and 21 metabolites. Further intersection with positively associated metabolites from our prior MR analysis examining the relationship between plasma metabolites and ASD yielded 4 common metabolites (Figure 4 and Supplementary Table S3). This allowed us to calculate specific MR effect estimates and *p*-values for the interactions among these three elements. Additionally, we quantified both the direct effects of the microbiota on ASD and the indirect effects mediated through metabolites. In conclusion, our findings illuminate that ASD’s genetic predisposition may exert its influence through alterations in plasma metabolites, thereby impacting ASD progression from a genetic standpoint.

## 4. Discussion

This study investigated the intricate relationship between gut microbiota, plasma metabolites, and Autism Spectrum Disorder (ASD), with a focus on the microbiota-metabolite-disease interplay. Through the integration of Linkage Disequilibrium Score Regression (LDSC) and Mendelian Randomization (MR) approaches, we identified significant associations between specific gut microbes, plasma metabolites, and ASD, while providing causal evidence to unravel their roles in ASD pathogenesis. These findings reveal the existence of a gut microbiota-plasma metabolite-ASD axis, offering new perspectives for understanding the biological mechanisms underlying ASD. Furthermore, this study highlights the potential of utilizing gut microbiota and plasma metabolites as biomarkers for ASD diagnosis and as targets for developing innovative therapeutic interventions.

Gut microbiota has been increasingly recognized as a key contributor in the etiology of ASD. Studies utilizing 16S rRNA sequencing and metagenomics have consistently demonstrated significant dysbiosis in ASD patients, characterized by a lack of the typical age-related increase in alpha diversity, reduced *Bacteroidota* abundance, and an elevated *Bacillota/Bacteroidota* ratio [10]. At the genus level, notable changes include reduced levels of *Alistipes*, *Bilophila*, and *Parabacteroides*, alongside increased levels of *Collinsella* and *Lactobacillus*, highlighting a strong link between gut microbiota and ASD [26], although causality has remained unclear. Specific bacterial taxa, such as *Lactobacillus*, *Bacillus* spp., and *Streptococcus* spp., have been hypothesized to influence ASD through the gut–brain axis, potentially via the production of neurotransmitters like serotonin and dopamine [27,28]. In our study, using the LDSC method, we identified a genetic negative correlation between the *Lachnospiraceae NK4A136 group*, *Sellimonas*, and ASD. The *Lachnospiraceae NK4A136 group* has been widely reported to be associated with gut inflammation [29], with high fecal expression levels observed in Chinese children with ASD [30] and potential regulatory roles identified in the VPA-induced ASD mouse model [31]. Similarly, *Sellimonas* has been found to decrease in abundance in psychiatric disorders [32], with fecal microbiota transplantation (FMT) in ASD mouse models further revealing its mechanistic link to ASD [33]. To further address causality, we employed MR analysis, which revealed a positive association between *Turicibacter* and ASD, while *Ruminococcus 1*, *Ruminiclostridium*, *Ruminococcaceae UCG-005*, *Sutterella*, and *Dorea* were negatively associated with ASD. *Turicibacter*, as identified in our previous 16P11.2^dp/+^ ASD mouse model, is hypothesized to impair gut barrier function and promote inflammation, both of which may contribute to ASD development [34,35]. In both human data and our mouse model, we observed lower expression levels of *Ruminococcus* and *Ruminiclostridium* in ASD [36]. Previous studies have also reported a significant reduction in *Dorea* and *Sutterella* in children with ASD and functional abdominal pain [37], although inconsistent findings have been noted, with some studies observing an increase in *Sutterella* [38], possibly due to the heterogeneity of ASD and variations in clinical characteristics. Our MR analysis provides causal evidence, indicating that *Sutterella* may genetically slow the progression of ASD. *RuminococcaceaeUCG-005*, known for its protective role against gut inflammation, has also been reported to exhibit a negative correlation with ASD [39], which aligns with our findings, shedding light on the complex interplay between gut microbiota and ASD and offering new insights into potential therapeutic targets for intervention.

Plasma metabolites also play a critical role in the pathophysiology of ASD. Children with ASD consistently exhibit abnormal metabolite concentrations, reflecting disruptions in key pathways such as lipid, amino acid, neurotransmitter, and vitamin metabolism [40]. These disturbances align with metabolic changes observed in acute stress disorder, suggesting that shared mechanisms—such as oxidative stress, chronic inflammation, impaired energy metabolism, and disrupted signal transduction—may underlie the pathophysiology of ASD by interfering with normal brain development and function [41]. For example, altered serotonin levels may influence the gut–brain axis and modulate neurodevelopmental processes, while disruptions in amino acid metabolism, including imbalances in glutamate and GABA pathways, may hinder neurotransmitter synthesis and brain development. Lipid metabolism abnormalities, such as altered levels of polyunsaturated fatty acids, have also been linked to neuronal membrane dysfunction and neuroinflammation, both of which are implicated in ASD pathogenesis. However, establishing the causal relationship between these metabolite changes and ASD remains challenging, partly due to the difficulties of directly measuring intracranial metabolites, which limits causal research. Through LDSC analysis, we identified 59 metabolites or metabolite ratios associated with ASD, and using bidirectional Mendelian Randomization (MR), we demonstrated that 56 plasma metabolites or metabolite ratios are causal factors for ASD. These metabolites span crucial pathways, including lipid metabolism (e.g., 3-hydroxyhexanoate, Docosatrienoate), amino acid metabolism (e.g., Threonate, Cystathionine), neurotransmitter metabolism (e.g., Serotonin, 3-methoxytyramine sulfate), and vitamin metabolism (e.g., Pyridoxate, Ascorbic acid 2-sulfate). Notably, several of these metabolites have been previously associated with cognitive and behavioral impairments in ASD, but their causal roles remained unclear until now. For example, serotonin and its downstream metabolites are well-recognized for their influence on the gut–brain axis [42], while pyridoxate and ascorbic acid 2-sulfate are essential for maintaining redox homeostasis and providing neuroprotection [43]. These findings provide the first robust causal evidence linking plasma metabolites to ASD, offering a deeper understanding of how metabolic disruptions contribute to neurodevelopmental disorders.

Metabolites are often regarded as passive intermediates, subject to regulation by disease states, environmental factors, and other external stimuli; however, gut microbiota actively modulate metabolite levels, which in turn mediate the effects of the microbiota on ASD and other neurological disorders, underscoring the importance of understanding the causal relationships within the gut microbiota-plasma metabolite-ASD axis. Previous studies indicated that *RuminococcaceaeUCG-005* may influence neurotransmitter synthesis through the production of short-chain fatty acids (SCFAs), while Dorea has been implicated in modulating serotonin and dopamine levels within the gut–brain axis [44]. To elucidate the causal relationships among gut microbiota, plasma metabolites, and ASD, we conducted mediation Mendelian Randomization (MR) analysis, which demonstrated that gut microbiota affect ASD risk by altering plasma metabolite levels. Specifically, *Ruminiclostridium5* was linked to changes in Delta-CEHC and Docosadioate (C22-DC), *RuminococcaceaeUCG-005* was associated with serotonin regulation, and *Sutterella* influenced the levels of N-acetyl-L-glutamine. The involvement of vitamin E, lipid metabolism, and neurotransmitters indicates that the regulation of ASD by gut microbiota likely occurs through multiple pathways. This Mendelian randomization study provides strong causal evidence for the gut microbiota–plasma metabolite–ASD axis, emphasizing its critical role in ASD pathogenesis. From a clinical-translational perspective, the specific microbial taxa and plasma metabolites identified—such as *RuminococcaceaeUCG-005* linked to C22-DC and serotonin and *Sutterella* associated with N-acetyl-L-glutamine—form a promising biomarker panel. This panel could enable early auxiliary diagnosis and subtype stratification for ASD, while the elucidated causal pathways highlight actionable targets for microbial intervention, laying a scientific foundation for future therapies involving probiotics, prebiotics, or dietary supplements.

Despite its strengths, this study has several limitations. First, the ASD GWAS data were derived solely from the Danish Psychiatric Central Research Register (DPCRR), limiting the generalizability of our findings to other populations. Second, our reliance on existing GWAS data may have excluded important metabolites or microbial traits, restricting the scope of causal effects. Third, while sensitivity analyses were conducted, horizontal pleiotropy cannot be completely ruled out, potentially biasing causal estimates. Additionally, our mediation MR analysis focused on single mediators, without assessing the interactions among multiple mediators, which may underestimate the complexity of the gut microbiota-metabolite-ASD pathway. Finally, despite a relatively large sample size, ASD’s complex pathophysiology likely involves additional unexplored pathways.

## 5. Conclusions

In this study, we comprehensively investigated the impact of gut microbiota and plasma metabolite levels on the progression of ASD. Initially, we identified both positive and negative causal relationships between gut microbiota, plasma metabolites, and ASD, and validated these findings using sensitivity analyses to ensure their robustness. Furthermore, we explored potential reverse causal relationships and ruled out any reverse causation of ASD on gut microbiota or plasma metabolites. The study identified three key bacterial taxa that influence ASD progression through four critical metabolites. These findings establish a causal link between gut microbiota and ASD and underscore the mediating role of specific plasma metabolites in this relationship.

## Figures and Tables

**Table 1:** The genetic correlations between gut microbes and ASD.

Trait1	Trait2	r_g_	SE	*p* Value
*Lachnospiraceae* NK4A136 group	ASD	−1.1956	0.4208	0.0045
*Sellimonas*	ASD	−0.4673	0.1666	0.0051

**Table 2:** Significant MR results of causal association between gut microbes and ASD. The method used for MR analyses is inverse variance weighted.

Expoure	Outcome	Beta ± SE	*p* Value	HR (95% CI)
*Turicibacter*	ASD	0.1310 ± 0.0630	0.0375	1.1400 (1.0076–1.2898)
*Ruminococcaceae* UCG005	ASD	−0.2538 ± 0.0749	7 × 10^−4^	0.7758 (0.6699–0.8984)
*Sutterella*	ASD	−0.1966 ± 0.0935	0.0356	0.8215 (0.6839–0.9868)
*Ruminiclostridium5*	ASD	−0.2077 ± 0.0856	0.0152	0.8125 (0.6870–0.9609)
*Ruminococcus1*	ASD	−0.1846 ± 0.08419	0.0284	0.8315 (0.7050–0.9807)
*Dorea*	ASD	−0.2098 ± 0.0855	0.0142	0.8108 (0.6856–0.9587)

**Table 3:** The causal relationship between ASD and gut microbes were using the IVW method.

Expoure	Outcome	Beta ± SE	*p* Value	HR (95% CI)
ASD	*Turicibacter*	0.0207± 0.2955	0.9443	1.0209 (0.5720–1.8219)
ASD	*Ruminococcaceae* UCG005	0.0521 ± 0.2215	0.8143	1.0534 (0.6824–1.6262)
ASD	*Sutterella*	−0.0561 ± 0.1573	0.7215	0.9455 (0.6946–1.2869)
ASD	*Ruminiclostridium5*	−0.0332 ± 0.2272	0.8839	0.9674 (0.6198–1.5100)
ASD	*Ruminococcus1*	−0.0962 ± 0.1039	0.3451	0.9082 (0.7410–1.1133)
ASD	*Dorea*	−0.0506 ± 0.1583	0.7495	0.9507 (0.6970–1.2967)

**Table 4:** The genetic correlations between plasma metabolites and ASD.

Trait1	Trait2	r_g_	SE	*p* Value
4-methyl-2-oxopentanoate	ASD	−0.6174	0.2172	0.0045
Alpha-hydroxyisocaproate	ASD	−0.6501	0.2365	0.0060
1-stearoyl-GPI(18:0)	ASD	−1.0537	0.4341	0.0152
4-acetylphenolsulfate	ASD	−0.9291	0.2787	0.0009
Indolepropionate	ASD	−0.3776	0.1522	0.0131
Pyridoxate	ASD	0.5543	0.2586	0.0321
Alpha-hydroxysovalerate	ASD	−0.3251	0.1199	0.067
Hexanoylglycine	ASD	1.7316	0.8041	0.0313
Taurocholenatesulfate	ASD	−0.2675	0.1187	0.0242
Palmitoylsphingomyelin(d18:1/16:0)	ASD	−0.2580	0.1256	0.0400
2s,3R-dihydroxybutyrate	ASD	−0.4529	0.1290	0.0004
1-(1-enyl-stearoyl)-GPE(p-18:0)	ASD	−0.2683	0.1357	0.0480
2-hydroxydecanoate	ASD	0.3812	0.1873	0.0418
2-aminophenolsulfate	ASD	0.7891	0.3992	0.0481
P-cresolglucuronide	ASD	0.2877	0.1087	0.0081
Lignoceroylsphingomyelin(d18:1/24:0)	ASD	−0.2622	0.1267	0.0385
2-hydroxybutyrate/2-hydroxysobutyrate	ASD	−0.4417	0.1758	0.0120
Tricosanoylsphingomyelin(d18:1/23:0)	ASD	−0.3056	0.1440	0.0338
1-(1-enyl-stearoyl)-2-arachidonoyl-GPE(p-18:0/20:4)	ASD	−0.6361	0.2698	0.0184
Caffeicacidsulfate	ASD	0.2972	0.1460	0.0418
Sphingomyelin(d18:2/24:2)	ASD	−0.2810	0.1215	0.0207
2-hydroxy-4-(methylthio)butanoicacid	ASD	0.2535	0.0986	0.0101
Pentoseacid	ASD	−0.7062	0.3562	0.0474
Pantothenate	ASD	0.2376	0.1172	0.0427
2-aminobutyrate	ASD	−0.4337	0.1736	00125
4-acetamidobutanoate	ASD	−0.2659	0.1127	0.0183
Cys-gly,oxidized	ASD	0.2285	0.1076	0.0337
Arachidonate(20:4n6)	ASD	−0.4844	0.2182	0.0264
3-(4-hydroxyphenyl)lactate	ASD	−0.3912	0.1423	0.0060
Taurine	ASD	−0.4340	0.1997	0.0297
Cytidine	ASD	−0.4656	0.2223	0.0362
Bilirubindegradationproduct,C17H18N2O4(3)	ASD	-0.8692	0.3925	0.0268
Glycinetopyridoxalratio	ASD	−0.2364	0.1127	0.0360
Citrullinetodimethylarginine(SDMA+ADMA)ratio	ASD	0.3116	0.1488	0.0363
Uridinetocytidineratio	ASD	0.3035	0.1473	0.0393
Pyruvateto3-methyl-2-oxobutyrateratio	ASD	0.2385	0.1204	0.0477
Cysteinylglycinetotaurineratio	ASD	0.9990	0.3513	0.0045
Salicylatetocaprylate(8:0)ratio	ASD	−2.8567	0.9801	0.0036
Glucosetosucroseratio	ASD	−1.2513	0.4655	0.0072
Fructosetosucroseratio	ASD	−0.5650	0.1929	0.0034
Caffeinetotheophyllineratio	ASD	0.5331	0.2424	0.0278
Alpha-ketobutyratetopyruvateratio	ASD	−0.5827	0.2642	0.0274
CytidinetoN-acetylneuraminateratio	ASD	-0.3967	0.1625	0.0146
CytidinetoN-acetylglucosaminetoN-acetylgalactosan	ASD	−1.7244	0.7071	0.0147
Arachidonate(20:4n6)toparaxanthineratio	ASD	−0.4253	0.1868	0.0228
Paraxanthinetolinoleate(18:2n6)ratio	ASD	0.2857	0.1313	0.0296
Salicylatetooxalate(ethanedioate)ratio	ASD	−0.9387	0.4150	0.0237
Alpha-ketobutyrateto3-methyl-2-oxovalerateratio	ASD	−0.5800	0.2943	0.0487
3-methyl-2-oxovalerateto4-methyl-2-oxopentanoate	ASD	0.5238	0.1758	0.0029

**Table 5:** Significant MR results of causal association between ASD and plasma metabolites were using the IVW method.

Expoure	Outcome	Beta ± SE	*p* Value	HR (95% CI)
ASD	9,10-DiHOME	0.0933 ± 0.0416	0.0248	1.0978 (1.0119–1.1910)
ASD	1beta-hydroxyetiocholanolone glucuronide	−0.1401 ± 0.0515	0.0065	0.8692 (0.7858–0.9616)

## Data Availability

The original contributions presented in this study are included in the article/Supplementary Materials. Further inquiries can be directed to the corresponding authors.

## Supplementary Materials

The following supporting information can be downloaded at: https://www.mdpi.com/article/10.3390/pathogens14111137/s1, Figure S1: Detailed overview of study design and methodology; Figure S2: Scatter plots for causal effects of gut microbes on ASD; Figure S3: Leave-one-out sensitivity analysis of gut microbes associated with ASD; Table S1: The causal relationship between plasma metabolites and ASD were using the IVW method; Table S2: The causal relationship between ASD and plasma metabolites were using the IVW method; Table S3: Statistical data from two-step Mendelian randomization analyses of the causal relationships between gut microbes, plasma metabolites, and ASD.
