# Supplementary material for: Gut Microbiotas, Plasma Metabolites, and Autism Spectrum Disorder: A Bidirectional Mendelian Randomization Analysis
Source: Pathogens. 2025 Nov 10;14(11):1137. doi: 10.3390/pathogens14111137 (PMC12655273; doi:10.3390/pathogens14111137)
Supplement: Supplementary file 1 [file pathogens-14-01137-s001.zip › pathogens-3865881-supplementary.pdf]

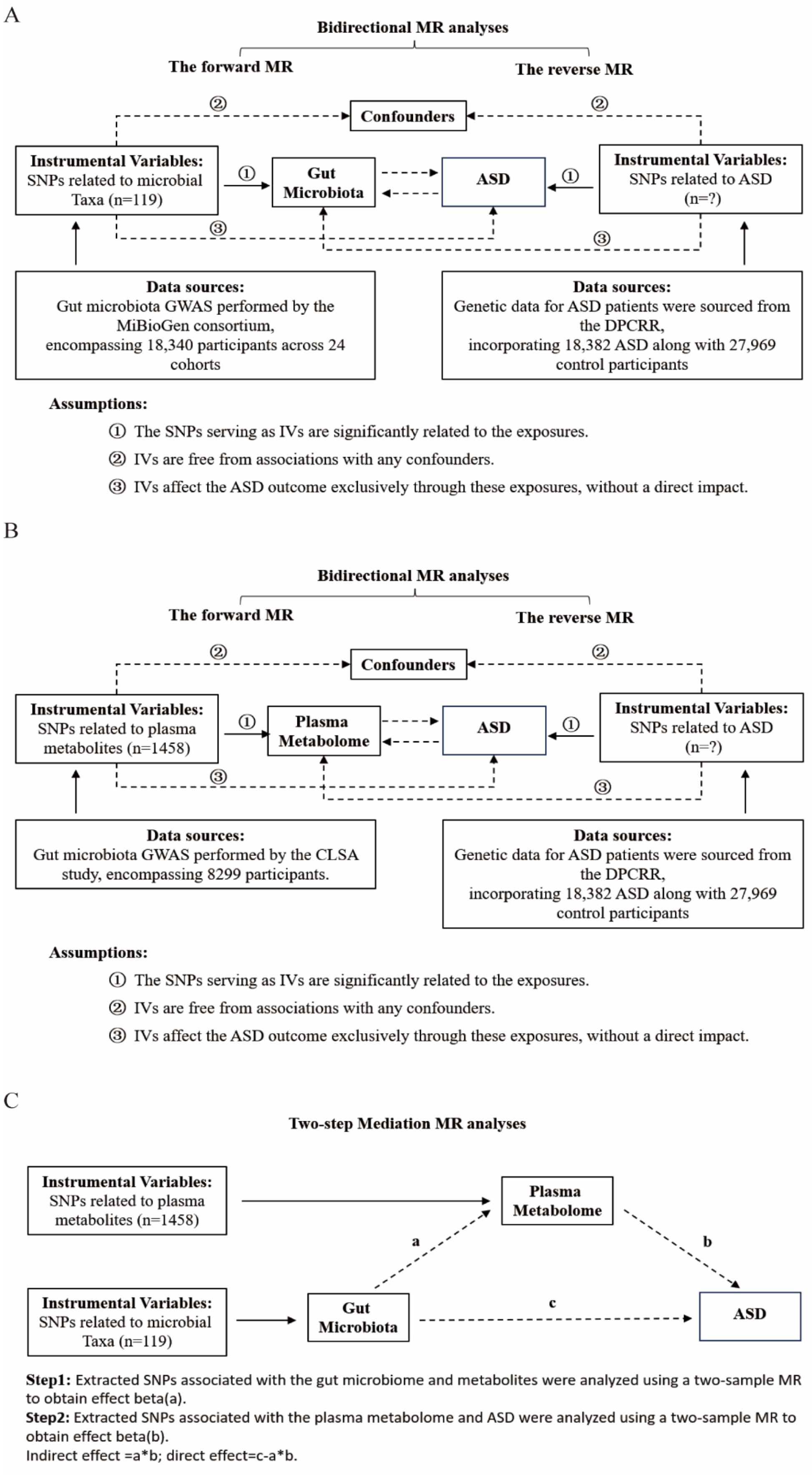


**Supplementary Figure S1.** Detailed overview of study design and methodology

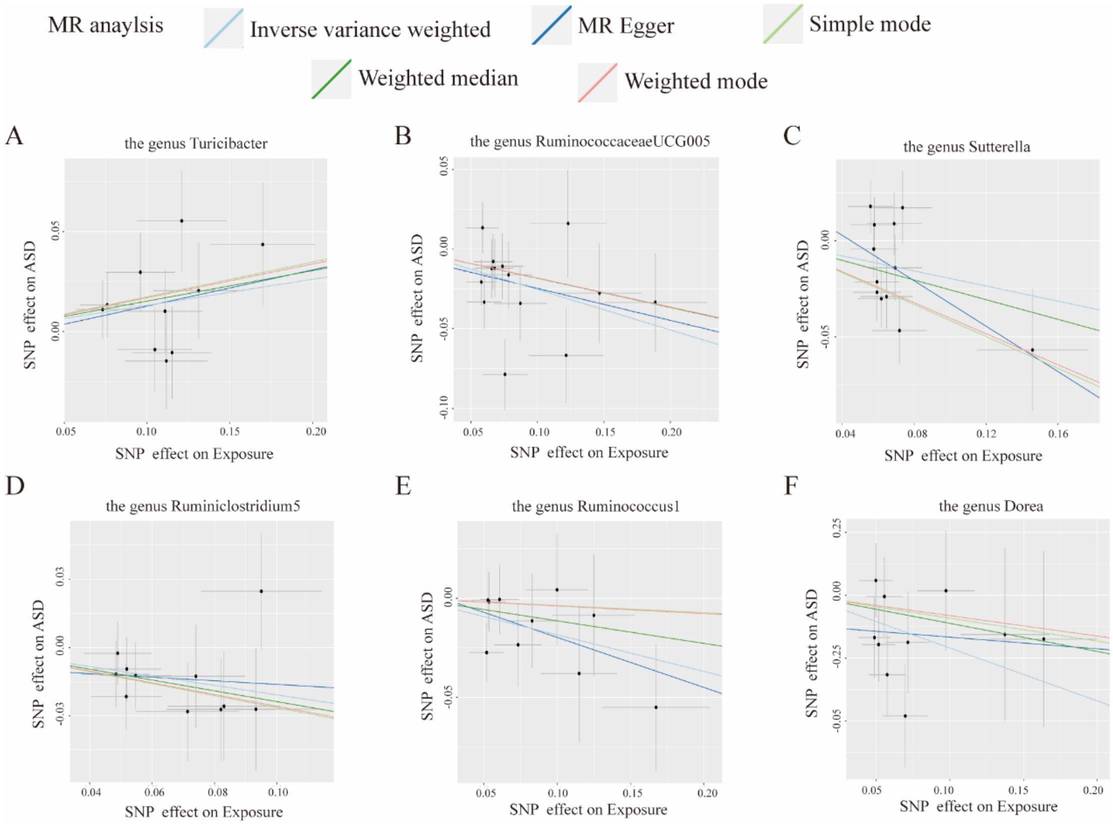


**Supplementary Figure S2.** Scatter plots for causal effects of gut microbes on ASD.

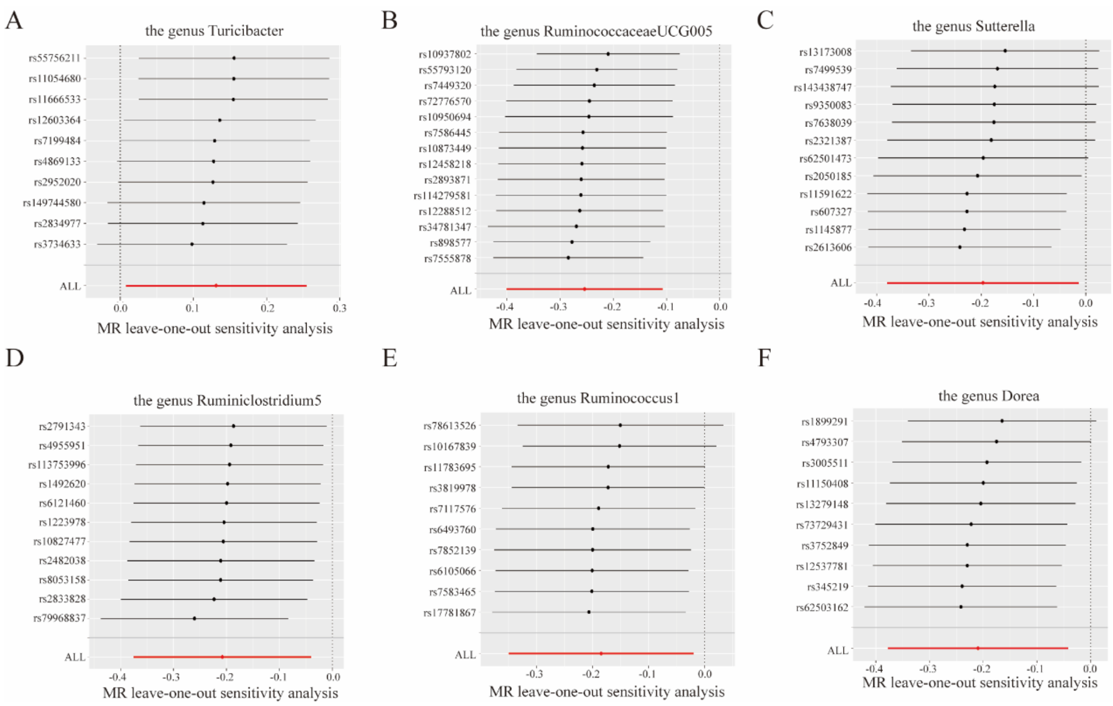


**Supplementary Figure S3.** Leave-one-out sensitivity analysis of gut microbes associated with ASD.

**SupplementaryTable S1.** The causal relationship between plasma metabolites and ASD were using the IVW method.

| Expoure | Outcome | Beta±SE | Pvalue | HR (95% CI) |
|---|---|---|---|---|
| 3-hydroxyoctanoate levels | ASD | -0.0959±0.0467 | 0.0401 | 0.9085(0.8290-0.9957) |
| 1-linoleoylglycerol (18:2) levels | ASD | -0.1273±0.0580 | 0.0282 | 0.8804(0.7857-0.9865) |
| Threonate levels | ASD | -0.1065±0.0518 | 0.0397 | 0.8989(0.8121-0.9950) |
| Galactonate levels | ASD | -0.1837±0.0712 | 0.0098 | 0.8322(0.7238-0.9568) |
| 5-hydroxyhexanoate levels | ASD | -0.1162±0.0437 | 0.0078 | 0.8903(0.8171-0.9699) |
| Docosatrienoate (22:3n3) levels | ASD | 0.0923±0.0453 | 0.0417 | 1.0968(1.0034-1.1988) |
| 1-palmitoyl-GPC (16:0) levels | ASD | -0.1048±0.0411 | 0.0109 | 0.9005(0.8306-0.9761) |
| Isovalerylcarnitine (C5) levels | ASD | -0.0937±0.0381 | 0.0139 | 0.9105(0.8450-0.9812) |
| Gamma-glutamyl-2-amino butyrate levels | ASD | 0.1057±0.0498 | 0.0339 | 1.1115(1.0080-1.2255) |
| Chiro-inositol levels | ASD | -0.1267±0.0492 | 0.0101 | 0.8810(0.8000-0.9703) |
| 5alpha-androstan-3alpha,17beta-diol disulfate levels | ASD | -0.1420±0.0422 | 0.0008 | 0.8676(0.7988-0.9424) |
| 2-oxoarginine levels | ASD | 0.1161±0.0507 | 0.022 | 1.1231(1.0169-1.2404) |
| Docosadioate (C22-DC) levels | ASD | 0.0999±0.0495 | 0.0435 | 1.1051(1.0029-1.2177) |
| Glycohyocholate levels | ASD | -0.1210±0.0487 | 0.0129 | 0.8860(0.8054-0.9747) |
| 3-methoxytyramine sulfate levels | ASD | -0.0870±0.0409 | 0.0335 | 0.9167(0.8460-0.9932) |
| Methionine sulfone levels | ASD | -0.0620±0.0254 | 0.0144 | 0.9398(0.8942-0.9877) |
| Sphingomyelin (d18:1/24:1, d18:2/24:0) levels | ASD | 0.1235±0.0537 | 0.0215 | 1.1314(1.0184-1.2571) |
| 3-methoxycatechol sulfate (2) levels | ASD | 0.1075±0.0526 | 0.0409 | 1.1135(1.0044-1.2344) |
| Sphingomyelin (d18:1/22:2, d18:2/22:1, d16:1/24:2) levels | ASD | -0.1015±0.0470 | 0.0310 | 0.9035(0.8240-0.9908) |
| Sphingomyelin (d17:1/16:0, d18:1/15:0, d16:1/17:0) levels | ASD | -0.0825±0.0349 | 0.0181 | 0.9208(0.8599-0.9860) |
| 4-hydroxyphenylacetoyl carnitine levels | ASD | 0.0830±0.0405 | 0.0407 | 1.0866(1.0035-1.1766) |
| 1-(1-enyl-palmitoyl)-2-palmitoyl-GPC (P-16:0/16:0) levels | ASD | -0.0553±0.0273 | 0.0430 | 0.9462(0.8968-0.9983) |
| Methylsuccinoylcarnitine levels | ASD | -0.0515±0.0250 | 0.0396 | 0.9498(0.9043-0.9976) |
| Ascorbic acid 2-sulfate levels | ASD | 0.1049±0.0455 | 0.0211 | 1.1106(1.0159-1.2141) |
| 4-hydroxyphenylacetylglutamine levels | ASD | 0.1003±0.0511 | 0.0498 | 1.1055(1.0001-1.2220) |
| Eicosenoylcarnitine (C20:1) levels | ASD | -0.0763±0.0378 | 0.0433 | 0.9265(0.8605-0.9977) |
| Dodecenedioate (C12:1-DC) levels | ASD | -0.1261±0.0429 | 0.0033 | 0.8815(0.8104-0.9588) |
| Hydroxyasparagine levels | ASD | 0.1153±0.0531 | 0.0301 | 1.1222(1.0112-1.2454) |
| Tetradecadienoate (14:2) levels | ASD | -0.1461±0.0630 | 0.0204 | 0.8641(0.7638-0.9776) |
| Delta-CEHC levels | ASD | 0.1067±0.0472 | 0.0239 | 1.1126(1.0142-1.2205) |
| Methyl vanillate sulfate levels | ASD | 0.1048±0.0351 | 0.0028 | 1.1105(1.0367-1.1895) |
| 11beta-hydroxyetiocholanolone glucuronide levels | ASD | -0.1351±0.0433 | 0.0018 | 0.8736(0.8026-0.9510) |
| 4-methylhexanoylglutamine levels | ASD | 0.1277±0.0504 | 0.0112 | 1.1363(1.0294-1.2542) |
| 5-hydroxymethyl-2-furoylcarnitine levels | ASD | -0.1356±0.0361 | 0.0002 | 0.8732(0.8136-0.9372) |
| Bilirubin degradation product, C16H18N2O5 (4) levels | ASD | -0.0496±0.0240 | 0.0389 | 0.9516(0.9078-0.9975) |
| Tetrahydrocortisol glucuronide levels | ASD | -0.1070±0.0419 | 0.0106 | 0.8986(0.8278-0.9754) |
| Methyl indole-3-acetate levels | ASD | -0.1174±0.0581 | 0.0434 | 0.8892(0.7934-0.9966) |
| Vanillylmandelate (VMA) levels | ASD | -0.0748±0.0377 | 0.0471 | 0.9279(0.8618-0.9990) |
| Anthranilate levels | ASD | -0.0781±0.0384 | 0.0420 | 0.9248(0.8578-0.9972) |
| Glycerol levels | ASD | -0.1298±0.0626 | 0.0381 | 0.8783(0.7769-0.9929) |
| 9,10-DiHOME levels | ASD | 0.1079±0.0492 | 0.0282 | 1.1139(1.0116-1.2266) |
| 3-Hydroxybutyrate levels | ASD | -0.1225±0.0618 | 0.0474 | 0.8847(0.7838-0.9986) |
| Cystathionine levels | ASD | -0.0853±0.0416 | 0.0404 | 0.9183(0.8463-0.9963) |
| Serotonin levels | ASD | 0.1114±0.0488 | 0.0224 | 1.1178(1.0159-1.2300) |
| N-acetyl-L-glutamine levels | ASD | 0.0414±0.0200 | 0.0388 | 1.0423(1.0021-1.0840) |
| Bilirubin (E,E) levels | ASD | -0.0425±0.0217 | 0.0499 | 0.9584(0.9185-1.0000) |
| N6,N6-dimethyllysine levels | ASD | -0.0445±0.0201 | 0.0270 | 0.9565(0.9195-0.9949) |
| Adenosine 5'-diphosphate (ADP) to Adenosine5'-monophosphate (AMP)ratio | ASD | -0.1172±0.0368 | 0.0013 | 0.8894(0.8281-0.9554) |
| Adenosine 5'-diphosphate (ADP) to tyrosine ratio | ASD | -0.0941±0.0356 | 0.0082 | 0.9102(0.8488-0.9760) |
| Adenosine 5'-monophosphate (AMP) to phenylalanine ratio | ASD | 0.1038±0.0482 | 0.0315 | 1.1093(1.0093-1.2193) |
| Phosphate to N-acetylneuraminate ratio | ASD | -0.1112±0.0428 | 0.0094 | 0.8948(0.8228-0.9731) |
| Glycolithocholate to glycolithocholate sulfate ratio | ASD | 0.0886±0.0359 | 0.0135 | 1.0926(1.0184-1.1722) |
| Adenosine 5'-monophosphate (AMP) to histidine ratio | ASD | 0.1325±0.0559 | 0.0178 | 1.1417(1.0232-1.2739) |
| Glycerol to sulfate ratio | ASD | -0.1041±0.0499 | 0.0368 | 0.9011(0.8173-0.9936) |
| Succinate to proline ratio | ASD | 0.1025±0.05170 | 0.0473 | 1.1080(1.0012-1.2261) |
| Adenosine 5'-diphosphate (ADP) to glutamine ratio | ASD | -0.0804±0.0341 | 0.0185 | 0.9227(0.8630-0.9866) |
| Adenosine 5'-monophosphate (AMP) to N-palmitoyl-sphingosine (d18:1 to 16:0) ratio | ASD | 0.1100±0.0430 | 0.0107 | 1.1163(1.0259-1.2147) |
| Proline to glutamate ratio | ASD | 0.1157±0.0498 | 0.0201 | 1.1227(1.0183-1.2377) |

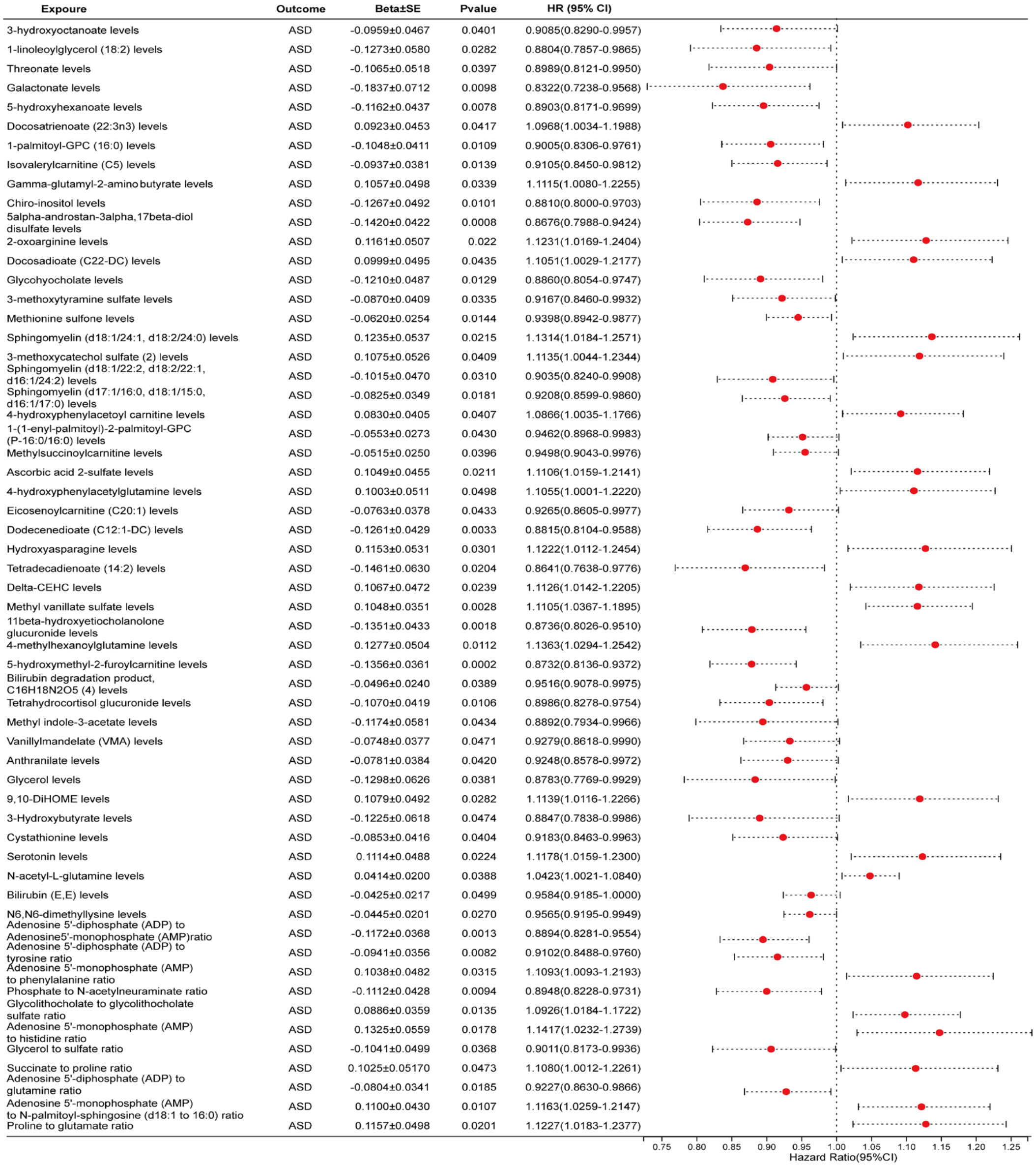

**Supplementary Table S2.** The causal relationship between ASD and plasma metabolites were using the IVW method.

| Exposure | Outcome | Beta±SE | Pvalue | HR (95% CI) |
|---|---|---|---|---|
| ASD | 3-hydroxyoctanoate | -0.0614±0.0391 | 0.1164 | 0.9404(0.871-1.0154) |
| ASD | 1-linoleoylglycerol (18:2) | -0.0633±0.0442 | 0.1522 | 0.9386(0.8607-1.0236) |
| ASD | Threonate | -0.0201±0.0398 | 0.6127 | 0.9801(0.9066-1.0595) |
| ASD | Galactonate | -0.0385±0.0414 | 0.3519 | 0.9622(0.8872-1.0435) |
| ASD | Pyridoxate | -0.0339±0.0479 | 0.4783 | 0.9666(0.8801-1.0617) |
| ASD | Docosatrienoate (22:3n3) | -0.0221±0.0461 | 0.6322 | 0.9782(0.8937-1.0707) |
| ASD | 1-palmitoyl-GPC (16:0) | -0.0225±0.0524 | 0.667 | 0.9777(0.8823-1.0835) |
| ASD | Isovalerylcarnitine (C5) | -0.0065±0.0383 | 0.8644 | 0.9935(0.9216-1.071) |
| ASD | Gamma-glutamyl-2-aminobutyrate | -0.0317±0.0477 | 0.5071 | 0.9688(0.8823-1.0639) |
| ASD | Androstenediol (3beta,17beta) disulfate | -0.1157±0.0594 | 0.0516 | 0.8907(0.7928-1.0008) |
| ASD | 5alpha-androstan-3alpha,17beta-diol disulfate | 0.0741±0.0421 | 0.0783 | 1.0769(0.9916-1.1696) |
| ASD | 2-oxoarginine | 0.023±0.0436 | 0.5976 | 1.0233(0.9395-1.1144) |
| ASD | Docosadioate (C22-DC) | 0.0672±0.0399 | 0.0921 | 1.0695(0.9891-1.1565) |
| ASD | Glycohyocholate | -0.0459±0.0445 | 0.3019 | 0.9551(0.8754-1.0421) |
| ASD | 3-methoxytyramine sulfate | -0.0298±0.0487 | 0.5396 | 0.9706(0.8823-1.0677) |
| ASD | N-formylanthranilic acid | -0.0423±0.0375 | 0.259 | 0.9586(0.8907-1.0316) |
| ASD | 3-methylglutarylcarnitine | 0.0266±0.0388 | 0.4938 | 1.0269(0.9517-1.1082) |
| ASD | 3-methoxycatechol sulfate | -0.07±0.0422 | 0.0973 | 0.9324(0.8583-1.0128) |
| ASD | 3-hydroxyhexanoate | -0.0045±0.036 | 0.9001 | 0.9955(0.9276-1.0683) |
| ASD | Sphingomyelin (d17:1/16:0, d18:1/15:0 , d16:1/17:0) | -0.0124±0.0383 | 0.7465 | 0.9877(0.9163-1.0647) |
| ASD | Glycodeoxycholate 3-sulfate | -0.0339±0.0407 | 0.4056 | 0.9667(0.8926-1.047) |
| ASD | 1-(1-enyl-palmitoyl)-2-palmitoyl-GPC (P-16:0/16:0) | -0.0108±0.0397 | 0.7865 | 0.9893(0.9152-1.0694) |
| ASD | (S)-3-hydroxybutyrylcarnitine | 0.001±0.0397 | 0.9794 | 1.001(0.9261-1.082) |
| ASD | Ascorbic acid 2-sulfate | -0.0472±0.0407 | 0.2456 | 0.9539(0.8808-1.033) |
| ASD | 4-hydroxyphenylacetylglutamine | -0.0084±0.0388 | 0.8278 | 0.9916(0.9191-1.0699) |
| ASD | Eicosenoylcarnitine (C20:1) | -0.0166±0.0449 | 0.711 | 0.9835(0.9007-1.0739) |
| ASD | Dodecenedioate (C12:1-DC) | -0.0369±0.0385 | 0.3378 | 0.9637(0.8936-1.0393) |
| ASD | Hydroxyasparagine | -0.0289±0.0377 | 0.4436 | 0.9715(0.9022-1.0461) |
| ASD | Tetradecadienoate (14:2) | -0.0339±0.038 | 0.3733 | 0.9667(0.8972-1.0415) |
| ASD | Delta-CEHC | 0.0244±0.0459 | 0.5945 | 1.0247(0.9366-1.1211) |
| ASD | Methyl vanillate sulfate | 0.0168±0.0554 | 0.7616 | 1.0169(0.9123-1.1336) |
| ASD | 11beta-hydroxyetiocholanolone glucuronide | -0.1401±0.0515 | 0.0065 | 0.8693(0.7858-0.9616) |
| ASD | 4-methylhexanoylglutamine | 0.0543±0.0477 | 0.2551 | 1.0558(0.9616-1.1592) |
| ASD | 5-hydroxymethyl-2-furoylcarnitine | -0.0193±0.0509 | 0.7046 | 0.9809(0.8878-1.0837) |
| ASD | Bilirubin degradation product, C17H20 N2O5 | -0.0398±0.0393 | 0.3114 | 0.961(0.8897-1.038) |
| ASD | Tetrahydrocortisol glucuronide | -0.0429±0.0383 | 0.2622 | 0.958(0.8888-1.0326) |
| ASD | Methyl indole-3-acetate | -0.0325±0.0483 | 0.5013 | 0.968(0.8806-1.0642) |
| ASD | Vanillylmandelate (VMA) | -0.0015±0.0349 | 0.9647 | 0.9985(0.9325-1.069) |
| ASD | Anthranilate | 0.0333±0.0477 | 0.4848 | 1.0339(0.9416-1.1351) |
| ASD | Glycerol | 0.0112±0.0385 | 0.7711 | 1.0112(0.9378-1.0904) |
| ASD | 9,10-DiHOME | 0.0933±0.0416 | 0.0248 | 1.0978(1.0119-1.191) |
| ASD | 3-Hydroxybutyrate | 0.0106±0.0395 | 0.7874 | 1.0107(0.9355-1.092) |
| ASD | Cystathionine | 0.0362±0.0396 | 0.3602 | 1.0369(0.9595-1.1206) |
| ASD | Serotonin | -0.0099±0.0421 | 0.8137 | 0.9901(0.9117-1.0753) |
| ASD | N-acetyl-L-glutamine | 0.0261±0.039 | 0.5034 | 1.0264(0.9509-1.1079) |
| ASD | Bilirubin | -0.0362±0.0394 | 0.358 | 0.9645(0.8929-1.0418) |
| ASD | N6,N6-dimethyllysine | 0.0121±0.0413 | 0.7688 | 1.0122(0.9334-1.0977) |
| ASD | Adenosine 5'-diphosphate (ADP) to Adenosine 5'-monophosphate (AMP) ratio | -0.0309±0.0538 | 0.5658 | 0.9696(0.8726-1.0774) |
| ASD | Adenosine 5'-diphosphate (ADP) to tryosine ratio | -0.0229±0.0535 | 0.669 | 0.9774(0.8802-1.0854) |
| ASD | Adenosine 5'-monophosphate (AMP) to phenylalanine ratio | -0.0141±0.0402 | 0.7251 | 0.986(0.9113-1.0667) |
| ASD | Phosphate to N-acetylneuraminate ratio | 0.0051±0.0376 | 0.8916 | 1.0051(0.9337-1.082) |
| ASD | Glycolithocholate to glycolithocholate sulfate ratio | 0.0197±0.0434 | 0.6494 | 1.0199(0.9367-1.1105) |
| ASD | Adenosine 5'-monophosphate (AMP) to histidine ratio | 0.0263±0.0428 | 0.539 | 1.0266(0.9441-1.1164) |
| ASD | Glycerol to sulfate ratio | 0.023±0.038 | 0.5449 | 1.0233(0.9498-1.1025) |
| ASD | Succinate to proline ratio | 0.0107±0.0392 | 0.7839 | 1.0108(0.9361-1.0915) |
| ASD | Adenosine 5'-diphosphate (ADP) to glutamine ratio | -0.0337±0.0533 | 0.5272 | 0.9669(0.871-1.0733) |
| ASD | Adenosine 5'-monophosphate (AMP) to N -palmitoyl-sphingosine (d18:1 to 16:0) ratio | 0.0062±0.0422 | 0.8824 | 1.0063(0.9264-1.093) |
| ASD | Proline to glutamate ratio | -0.0229±0.0463 | 0.621 | 0.9773(0.8925-1.0703) |

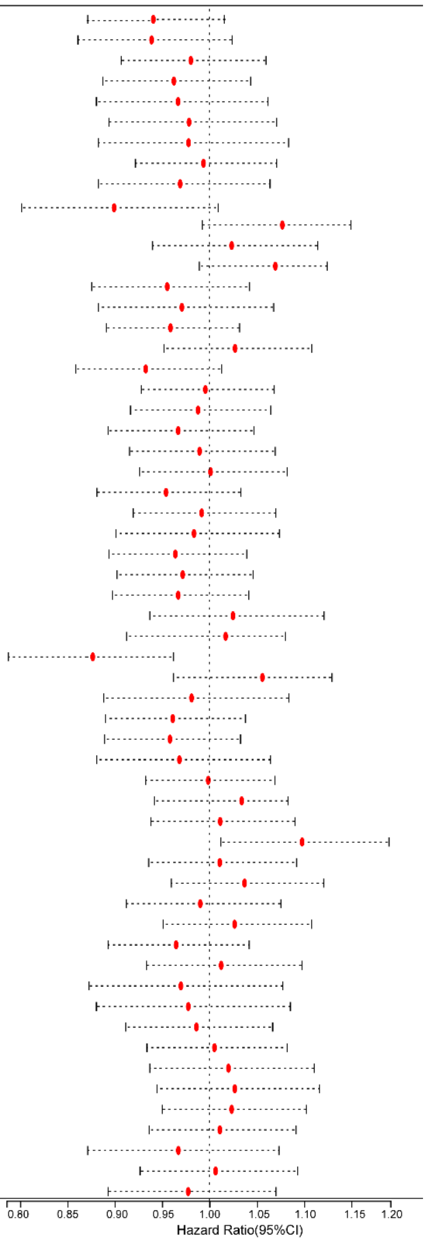


**Supplementary Table S3.** Statistical data from two-step Mendelian randomization analyses of the causal relationships between gut microbes, plasma metabolites, and ASD.

| Exposure | Mediator | Outcome | $\beta_{FM}$±SE | $\beta_{MO}$±SE | $\beta_{FO}$±SE | Pvalue | Direct effect | Indirect effect |
|---|---|---|---|---|---|---|---|---|
| Ruminiclostridium5 | Delta-CEHC levels | ASD | -0.2186 ±0.1038 | 0.1067 ±0.0472 | -0.2076 ±0.0856 | 0.0352 | -0.1843 | -0.0233 |
| Ruminiclostridium5 | Docosadioate (C22-DC) levels | ASD | -0.1886 ±0.0947 | 0.0999 ±0.0495 | -0.2076 ±0.0856 | 0.0463 | -0.1888 | -0.0188 |
| RuminococcaceaeUCG005 | Serotonin levels | ASD | -0.1730 ±0.0751 | 0.1114 ±0.0488 | -0.2538 ±0.0749 | 0.0213 | -0.2346 | -0.0193 |
| Sutterella | N-acetyl-L-glutamine levels | ASD | -0.1839 ±0.0862 | 0.0414 ±0.0200 | -0.1966 ±0.0935 | 0.033 | -0.189 | -0.0076 |
